# Personalized Medicine and Molecular Interaction Networks in Amyotrophic Lateral Sclerosis (ALS): Current Knowledge

**DOI:** 10.3390/jpm8040044

**Published:** 2018-12-13

**Authors:** Stephen Morgan, Stephanie Duguez, William Duddy

**Affiliations:** Northern Ireland Centre for Stratified Medicine, Altnagelvin Hospital Campus, Ulster University, Londonderry BT47 6SB, Northern Ireland, UK; Morgan-S20@ulster.ac.uk (S.M.); s.duguez@ulster.ac.uk (S.D.)

**Keywords:** ALS, personalized medicine, molecular interaction networks

## Abstract

Multiple genes and mechanisms of pathophysiology have been implicated in amyotrophic lateral sclerosis (ALS), suggesting it is a complex systemic disease. With this in mind, applying personalized medicine (PM) approaches to tailor treatment pipelines for ALS patients may be necessary. The modelling and analysis of molecular interaction networks could represent valuable resources in defining ALS-associated pathways and discovering novel therapeutic targets. Here we review existing omics datasets and analytical approaches, in order to consider how molecular interaction networks could improve our understanding of the molecular pathophysiology of this fatal neuromuscular disorder.

## 1. Introduction

Amyotrophic lateral sclerosis (ALS), also commonly known as Lou Gehrig’s disease, is a progressive neuromuscular disorder. ALS is the most common form of motor neuron disease (MND) and is characterised by progressive degeneration of both upper and lower motor neurons. Symptoms associated with the disease include muscle atrophy, limb paralysis, dysphagia, dysarthria, and respiratory failure. The annual incidence rate of ALS in the European population is approximately 2–3 per 100,000 people [1], with the highest incidence occurring in individuals >55 years of age. Prognosis is poor, with median survival time from onset to death of around 20–48 months [2]. ALS can be divided broadly into two categories, familial ALS (fALS) involving patients with a family history of disease, and sporadic ALS (sALS), involving patients with no previous family history. Cases are split approximately 90%–95% sALS and 5%–10% fALS [3,4]. 

With improving technologies in genomic analysis and large-scale international collaborations, the understanding of genetic factors in fALS has improved significantly [5]. Studies have been able to identify multiple ALS-associated genes, and from the discovery of the first ALS-associated gene *SOD1*, more than 20 genes have been attributed to the disease [6,7]. Pathogenic mutations have been identified in genes such as *ALS2*, *SETX*, *SPG11*, *FUS*, *VAPB*, *ANG*, *TARDBP*, *FIG4*, *OPTN*, *VCP*, *UBQLN2*, and *C9orf72* [8,9]. Multiple cell types have been postulated to play a role in pathology, such as muscle satellite cells, mitochondria, astrocytes, and microglia [10,11,12,13].

Riluzole and edaravone are currently the only two approved drug therapeutics available in the treatment of ALS and are observed to have a modest efficacy. Clinical trials involving riluzole treatment in ALS populations have indicated an improvement in survival of ALS patients compared to placebo controls [14,15]. Subsequent reviews have identified that riluzole can extend the median survival time of ALS patients by around 2–3 months [16]. The cost effectiveness of the drug, as well as the ability to treat the functional consequences of disease, such as muscle strength and bulbar or limb function, have been extensively studied [17]. Clinical trials involving edaravone treatment in ALS populations were originally unable to identify any statistically significant benefit between treatment groups and placebo groups [18]. Post hoc analysis subsequently identified a subpopulation of ALS patients with well-defined early stage ALS that appeared to respond to treatment with a reduction in the deterioration of their ALS Functional Rating score (ALSFRS-R) compared to placebo [19,20]. The efficacy of edaravone was only apparent in a subset of ALS patients with early stage ALS, and it is believed that any benefits observed would not translate to the whole ALS population [21]. The overall limited efficacy of both these approved ALS drugs highlights the necessity of developing more effective therapeutics.

Biomarkers are laboratory-measurable biological characteristics that can be attributed to a certain physiological or pathological process. Within ALS, the early diagnosis of patients remains difficult due to the phenotypic overlap between the disease and various oer motor neuron diseases. Biomarkers have the potential to provide more accurate diagnoses of ALS patients and to help stratify the ALS patient population into groups of responders to therapeutics, to act as prognostic markers in disease progression, and to lead us toward possible drug targets. The value of biomarkers in ALS has been demonstrated in previous publications. For example, studies have shown that cerebrospinal fluid (CSF) diagnostic assays can provide a suitable diagnostic tool for distinguishing ALS patients from control groups in their respective study populations. A study involving 41 ALS patients and 33 neurological disease controls was able to define a diagnostic assay with 87.5% sensitivity and 91.2% specificity, using the top five differentially expressed proteins between the two test groups (IL-10, IL-6, GM-CSF, IL-2, and IL-15) [22]. A separate study identified phosphorylated neurofilament heavy chain and complement C3 (pNFH/C3) as optimal biomarkers for a diagnostic assay when measuring levels of cytoskeletal proteins and inflammatory markers in CSF samples of ALS subjects, disease controls, and healthy controls. The diagnostic assay was able to identify ALS patients with 87.3% sensitivity and 94.6% specificity when applied to a study population consisting of 71 ALS subjects, 52 disease controls, and 40 healthy subjects [23]. However, CSF samples are relatively invasive and expensive to obtain compared to blood assays.

Multiple prognostic biomarkers have been identified to predict disease severity, originating from blood, muscle, CSF, and genetic factors [24]. Higher pNFH levels in plasma, serum, and CSF samples have been associated with a faster decline in the ALSFRS-R score, suggesting a more rapid disease progression [25]. Multiple inflammatory markers have been suggested to provide viable prognostic markers in ALS. Increased levels of wide-range C-reactive protein (wrCRP) [26], high mobility group box 1 (HMGB1) autoantibody, increased granzyme B, and increased CSF IL-8 levels [22,27,28] have all been implicated in correlating with a more rapid decline in the ALSFRS-R score. The number of genetic alterations associated with ALS continues to grow. Prognostically, the presence of some genetic variants indicates susceptibility to and increased severity of ALS. The most frequently known genetic cause of ALS is the hexanucleotide repeat (GGGGCC) expansion in the first intron of *C9ORF72* [22]. Patients exhibiting this repeat expansion are characterised by a lower age of onset and a shorter survival time [29,30]. Numerous other genetic variants have been associated with reduced survival in the ALS population, including *SLC11A2*, *UNC13A*, and *ZNF512B*, among many others [31,32,33]. Whereas prognostic and diagnostic biomarkers have been identified in ALS, their usefulness in terms of directing drug development is currently minimal.

Research investigating the molecular basis of ALS aims to elucidate molecular mechanisms involved in disease pathology with the hope of yielding insights into defining viable therapeutic targets. In ALS, multiple molecular mechanisms have been suggested. Glutamate excitotoxicity is believed to cause neuronal death either via abnormally high presynaptic neuron activity, impaired response to excitatory stimuli in the postsynaptic neuron, or a combination of both [34], and is believed to be a therapeutic target of riluzole. Examples include increased extracellular glutamate levels due to reduced clearance via glutamate transporters such as the excitatory amino acid transporter 2 (*EAAT2*) expressed on astrocytes [35,36], or increased synaptic release of glutamate via endoplasmic reticulum stress (ER) [37]. Mitochondrial dysfunction and death is considered an important component in ALS pathogenesis. Super oxide dismutase (*SOD1*) mutations have been observed to result in the accumulation of mutant SOD1 aggregates in mitochondria, causing mitochondrial damage and subsequent motor neuron death [38,39]. Morphological abnormalities in mitochondria and the presence of fusion and fission proteins have all been documented to disrupt normal mitochondria function in ALS [40]. Reactive oxygen species (*ROS*) accumulation has been linked to ALS due to reduced clearance by mutant SOD1 [41]. Protein toxicities in the form of TAR DNA-binding protein 43 (TDP-43), fused in sarcoma (FUS), optineurin (OPTN), and ubiquilin-2 (UBQLN2) aggregate in motor neuron and glial cells [42,43,44,45,46]. This list of mechanisms implicated in ALS is far from exhaustive, and many more mechanisms have been suggested. Considering the multitude of genes identified as associated with fALS and the varying molecular mechanisms described, ALS in many cases may be a multigenic systemic disease. As a result, applying a personalized approach to molecular data could provide a benefit to ALS patients if their specific mechanism of disease can be determined and therapies relevant to these disease mechanisms can be applied. The use of molecular interaction networks can aid in the understanding of disease mechanisms associated with ALS and can identify genes and biological pathways that may be perturbed by these mechanisms, to provide possible therapeutic targets.

Personalized medicine is the concept of tailoring treatment to the individual patient. With advancements in “omics” technologies (e.g., genome, proteome, metabolome), the ability to comprehensively define an individual’s omics profile, has illustrated how biologically heterogeneous we are [47]. Understanding how a person’s omics profile can influence their disease phenotype, response to treatment, and prognosis can allow clinicians to identify the most efficient and beneficial treatment plan for patients. Heritable factors and the variability of the underlying cause of disease can attribute to varying drug responses observed in patient populations [48].

An example of how personalised medicine approaches at the molecular level can be implemented to improve patient care is in cystic fibrosis (CF). Mutations in the CF transmembrane conductance regulator (*CFTR*) gene have been identified as the root cause for the disease [49,50,51]. Mutations in the *CFTR* gene result in a dysfunctional CFTR protein. In the airway epithelial cells, this results in viscous mucous secretions due to inadequate chloride transport. This results in chronic infection and inflammation, leading to respiratory failure [52]. In CF, there are currently three approved drugs that target specific genetic mutations in the CF population. Ivacaftor targets patients with at least one allele of the *G551D CFTR* gating mutation [53], lumacaftor/ivacaftor and tezacaftor/ivacaftor are combination treatments in the treatment of the most common *CFTR* mutation, Phe508del [54,55,56]. This example highlights the potential benefit of treating patients based on their personal genomic profile. Although the treatments are not generalizable across the whole population, they are able to produce significant benefits to the strata of patients they target [57,58,59]. Since many genes are implicated in ALS, a single therapeutic target to treat the whole patient population may not be feasible. With this in mind, a personalized medicine approach may be needed to effectively develop therapeutics for the ALS population.

**Figure 1:**
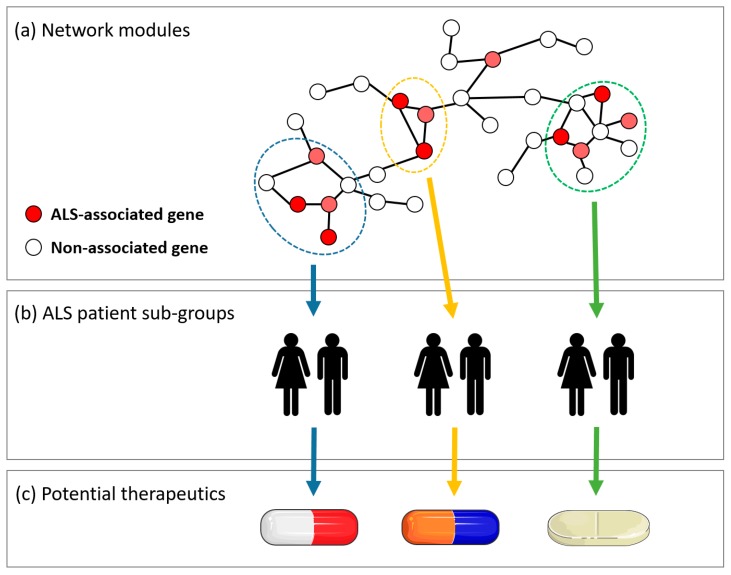
A central concept in the analysis of molecular interaction networks (MINs) is the identification of network modules, or clusters, of interactions that represent a process or pathway relevant to disease. The affected network module may differ between subgroups of a patient population, which could be used to develop personalized medicine approaches. (**a**) A miniature example of a MIN, with three network modules indicated in blue, green, and yellow. Within each module, specific genetic variants or biomolecules, indicated in red, are known to be associated with amyotrophic lateral sclerosis (ALS) pathology. (**b**) ALS subpopulations separated according to which module is affected or dysregulated. (**c**) Potential drug therapies can be tailored to target specific mechanisms within the ALS disease pathway, corresponding to specific patient subpopulations.

Within living organisms, a single protein or other biomolecule will rarely act alone to effect a given function. Instead, there is a complex series of interactions between multiple biomolecules that contributes to a biological process [60]. These interactions can include protein-protein binding, gene co-expression, RNA interactions, and many other types of molecular functional association. Functional associations can be represented using molecular interaction networks (MINs), with nodes denoting molecules and edges denoting the interactions between nodes. Biological networks have been identified as scale-free networks that follow a power law distribution [61]. This indicates that biological networks are not randomly connected, but instead exhibit a definite architecture related to biological processes contained within the interactions. Studying the structure and topology of MINs can help identify biomolecules that are involved in biological processes and elucidate which biomolecules or processes are dysfunctional in disease [62,63,64]. Identifying disease-implicated modules in networks will aid in narrowing the search for effective drug targets or disease biomarkers [65]. With a network view of a disease phenotype, multitarget therapies can be implemented to target dysfunctional processes that may be impractical to resolve with a single drug therapeutic [66]. Since ALS may in many cases be a multigenic complex systemic disease, MIN approaches are particularly relevant, as they could potentially identify one or more network clusters that are consistently associated with pathology but within which only a smaller subset of genes or other biomolecules are affected in any one patient (Figure 1).

In this review, we highlight the current applications of MINs in ALS and how further approaches might be applied toward personalized medicine in future analyses.

## 2. Omics Data in Amyotrophic Lateral Sclerosis

It would be impractical to review all of the many available omics datasets relevant to ALS, but here we examine a few key studies.

### 2.1. Transcriptomic

The Gene Expression Omnibus (GEO, http://www.ncbi.nlm.nih.gov/geo/) and the ArrayExpress Archive (http://www.ebi.ac.uk/arrayexpress) are international repositories for functional genomic data including microarrays, next-generation sequencing, or other high-throughput methods [67,68]. A search of the terms “ALS” or “amyotrophic lateral sclerosis” revealed more than 117 human studies of gene expression data in GEO, and 131 in ArrayExpress. These transcriptomic studies have shown, among other things, differing profiles between ALS patients, disease mimic syndromes, and healthy controls, and have enabled comparisons of transcriptomic profiles within ALS variations. One such study compared *C9ORF72* ALS frontotemporal dementia (FTD) patients to sporadic ALS/FTD patients. The results suggested that between these two variations of ALS, the transcriptomic profiles were comparatively similar, suggesting a common mechanism of disease [69]. This study highlights the potential for application of personalized medicine in ALS, as varying forms of the disease are being defined and analysed to determine mechanistic differences, or in this case similarities. Furthermore, by understanding different subtypes of ALS and their mechanistic underpinnings, it provides the opportunity for the development of subtype-specific therapies. Of particular relevance to MIN approaches, gene expression data can be represented as coexpression networks. Identifying modules of genes that are coexpressed can be suggestive of genetic interactions at a functional level [70,71]. Applications of gene coexpression network analysis have been successfully applied in ALS. Multiple gene coexpression studies have revealed several pathways believed to be implicated in ALS, including cell adhesion, calcium ion binding, inflammatory processes, and tumour necrosis factor (TNF) signalling [72,73,74,75,76,77]. Brohawn et al. took gene expression data gathered from seven ALS spinal cord samples and eight control spinal cord samples [78]. A post-filtered list of expressed genes was forwarded for weighted gene coexpression network analysis (WGCNA), which can be used to generate a gene coexpression network and to predict modules of highly associated genes that can be mapped to phenotypic traits. The authors were able to identify a module of coexpressed genes that was significantly associated with the ALS phenotype. They identified the hub genes present in this module and, with supplementary data, were able to select a gene, *TNFAIP2*, that when put forward for molecular testing appeared to induce motor neuron death when overexpressed. A limitation in this type of analysis is determining whether the gene coexpression module represents a causative factor in pathology or a consequence of downstream dysregulated physiological processes.

### 2.2. Genomic

In terms of understanding disease, genome wide association studies (GWAS) are used to identify functional genetic variants in disease populations that are not observed, or are observed less frequently, in the genomes of healthy controls. The National Human Genome Research Institute (NHGRI) GWAS Catalog (https://www.ebi.ac.uk/gwas) is a publicly available database of manually curated GWAS published data. It provides an interface that allows users to filter for gene-specific or condition-specific genetic variant associations [79]. From the GWAS Catalog resource, a list of ALS-associated genetic variants can be extracted: It currently contains >300 ALS-associated genetic variants. In addition, the ALS Online Database (ALSoD at http://alsod.iop.kcl.ac.uk) is a freely available resource containing genetic variant associations relevant to ALS only [80]. From the ALSoD, various lists of ALS-associated genetic variants can be retrieved, including lists of subpopulation genetic variant frequencies compared to the 1000 genomes reference. Studies involving a GWAS have the purpose of identifying potential biomarkers in ALS that may provide greater insight into disease pathology. A recent study of 20,806 ALS cases and 59,804 controls identified *KIF5A* as an ALS-associated gene [81] One study based on 250 sALS cases and 250 control cases from Chinese Han populations (CHP) was able to confirm the presence of five previously reported genetic variant loci present in other ALS populations as well as identifying eight novel genetic variant loci. [81,82,83]. The inclusion of genetic variants into analyses of MINs could help to identify common pathways in ALS or detect novel genetic associations to a known disease mechanism.

### 2.3. Proteomic

At the time of writing, the Proteomics Identification (PRIDE) Archive database (https://www.ebi.ac.uk/pride/archive) [84] included 10 Proteomic studies of ALS involving human samples. One study identified 806 proteins that were significantly reduced in *C9ORF72* ALS-induced pluripotent stem cells (iPSCs) compared to control iPSCs [85]. Gene ontology enrichment analysis of these proteins suggested abnormalities in oxidative phosphorylation and mitochondrial ribosomes. Another study specifically identified the protein-protein interaction (PPI) network of Matrin 3 (MATR3), a protein known to be associated with ALS. They were able to identify a deletion of the RNA recognition motif (RRM1) on MATR3 that diminished the binding of a subset of MATR3 binding proteins and was associated with the formation of intranuclear spherical structures in live cells [86]. The definition of MATR3 protein interactions provides increased coverage for PPI network databases, which can be used for MIN approaches. ALS studies comparing proteomic data between control and disease cohorts have been used to identify potential biomarkers, mainly based on analysis of the cerebral spinal fluid (CSF). These studies have been able to suggest biomarker panels for ALS diagnosis via CSF [87,88]. PPI databases such as STRING (https://string-db.org), BioGRID (https://thebiogrid.org), and IntAct (https://www.ebi.ac.uk/intact) all contain data linking interacting proteins [89,90,91] that can be used for MIN analyses. An example of this in ALS was a study in which differentially expressed genes (DEGs) between *C9ORF72* and control samples were first identified, and then a PPI was constructed based on the proteins encoded by these genes, using several PPI databases to define the pathways involved in the DEGs [77]. The authors were able to identify hub nodes within this network and define gene ontology (GO) terms believed to play a role in *C9ORF72* ALS pathophysiology, including cell adhesion, biological adhesion, and cell-cell signalling processes.

### 2.4. Metabolomic

The Human Metabolome Database (HMDB) (http://www.hmdb.ca) provides an interface to access a curated list of metabolites known to be abnormal in one or more specific diseases [92]. When searching for “ALS”, the database yields seven metabolites that have been observed to be abnormal in ALS patients: Homocysteine, L-thyronine, manganese, nitrate, picolinic acid, prostaglandin E2, and quinolinic acid [93,94,95,96,97,98]. A function of metabolomic data in terms of MINs could be in the construction of an interactome network representing all biomolecules and their interactions in a given cell or tissue type. In terms of personalized medicine, the use of metabolomics could help in the diagnosis and prognosis of ALS patients, for example by identifying biomarkers.

## 3. Molecular Data and Analyses Applied to the Study of Motor Neurons

A critical event in the pathology of ALS is the death of motor neurons (MNs). In this field, there is a developing debate as to whether motor neuron degeneration is a cell-autonomous process or if it depends on pathologic processes emerging in other cell and tissue types. The dying forward hypothesis suggests ALS pathology initiates MN degeneration, and any subsequent muscle atrophy is a downstream effect [99,100], whereas the dying back hypothesis suggests initiating pathology occurs at the muscle and neuromuscular junction, and subsequent motor neuron degeneration is a downstream effect [101]. Regardless of the direction of mechanism of pathology in ALS, motor neurons are pivotal in the disease mechanism and have the potential to provide biomarkers of the disease.

Transcriptomic analysis has been applied in many studies involving motor neurons, mainly to determine DEGs between control and disease cohorts. Examples of studies implementing transcriptomic analysis and MINs concurrently are less frequent. One such study compared the gene coexpression profiles of the ALS model of spinal motor neurons (spMN, derived from iPSCs) to fetal spinal tissues and adult spinal tissues [102]. The study was able to identify that the gene coexpression profile of the spMN more closely matched the fetal spMN than adult spMNs. This may indicate that to develop a more precise ALS model, the maturation and aging process needs to be replicated. Another study involving gene coexpression, specifically in the *C9ORF72* GGGGCC-expanded repeat mutation population, was able to identify coexpressed modules of genes that were differentially expressed between control and ALS MNs. The study was able to define six statistically significant GO terms associated with *C9ORF72* ALS pathophysiology, including the cholesterol biosynthetic process and the regulation-of-glucose metabolic process [75]. The authors noted that these findings support the involvement of endoplasmic reticulum (ER) stress, a process previously implicated in ALS. The ER is responsible for the correct folding of proteins, and aggregations of misfolded proteins are often observed in ALS [103].

GWA studies have led to the association of many genetic variants associated with motor neuron dysfunction in ALS. Mutations in the fused in sarcoma/translated in liposarcoma (*FUS/TLS*) gene on chromosome 16 in fALS [44] are believed to contribute to toxic protein aggregations in neuronal cytoplasm, similarly to the mechanism exhibited by mutant *SOD1* protein aggregates in ALS [104,105,106]. Mutations in the *TARDBP* gene that encodes the protein *TDP-43* have been identified in fALS families, with *TDP-43* aggregation observed in upper and lower motor neurons [107,108,109,110]. An application not yet explored for ALS genetic variant data is to include these variants into MINs such as gene coexpression or PPI networks. Examples of how this approach could be beneficial include a study of Alzheimer’s disease in which genetic variants were incorporated into a tissue-specific PPI network [111]. This revealed a strong indirect connection between large numbers of proteins from known AD loci, indicating a common pathological pathway. Furthermore, a study taking the genetic variants identified in sporadic autism cases was able to identify a large interconnected hub of genetic variant-associated proteins [112]. Of the 126 listed autism-related variants, 49 connected at a high degree within the PPI network. Downstream analysis of this highly connected cluster was able to identify a previously implemented pathway involving beta-catenin.

In ALS, the majority of proteomic studies of the motor neurons have involved the analysis and comparison of the proteome of the CSF between disease and control. All proteins accumulating in CSF are not necessarily specific to upper motor neuron tissue. The accumulation of neurofilaments in CSF is suggestive of motor neuron degradation [113,114]. Differential neurofilament markers between control and disease have been outlined as potential diagnostic biomarkers [85,86,87]. In a clinical setting, these biomarker panels may be of limited use for diagnostics due to the presence of ALS mimic phenotypes. A study was able to identify extremely high levels of CSF neurofilaments as suggestive of ALS, but not conclusively so [115]. Such data could be used in MIN analyses of PPI networks. A study compared the DEGs from brain biopsies in schizophrenia, bipolar disorder, and major depression [116]. These DEGs were mapped to a PPI network to characterize mutual protein interactions associated with these diseases. An approach such as this in ALS would be beneficial to clearly understand any differential pathology at the motor neuron level between ALS and ALS mimic conditions.

Metabolomic analyses in ALS, similarly to the study of the proteome, have been focused on the CSF rather than MN cells. The study of the metabolome between control and ALS populations has potential diagnostic value [117,118]. One study was able to identify a diagnostic profile for ALS from differential metabolite quantities compared to controls, which performed reasonably well (78.9% sensitivity, 76.5% specificity) in characterizing a test cohort [118]. Whether these metabolite markers would be generalizable across ALS populations is yet to be seen. Specific metabolome data on MNs in ALS is currently lacking, and with improving coverage of metabolomics pathways, MN-specific analysis may prove beneficial in dysfunctional pathway identification.

## 4. Molecular Data and Analyses Applied to Other ALS-Related Tissues and Cellular Compartments

### 4.1. Muscle

Muscle loss of function in ALS leads to muscle atrophy and subsequent mortality, generally due to respiratory failure. Evidence of muscle involvement as an instigating role in pathology has emerged. Studies of an ALS mouse model containing the *SOD1*^G93A^ mutation, selectively expressed in skeletal muscle, exhibited muscle atrophy prior to any visible MN degradation [119,120]. Furthermore, Nogo-A, a protein encoded by the *RTN4* gene, is believed to inhibit neurite outgrowth and MN regeneration [121]. Studies have identified a correlation between Nogo-A levels expressed in skeletal muscle and neuromuscular junction (NMJ) denervation in early ALS disease [122,123]. The role of muscle in ALS may be more than a victim. Understanding mechanisms involved in muscle atrophy in ALS could provide biomarkers and new therapeutic targets.

Gene expression analysis of skeletal muscle in ALS has identified numerous DEGs between control and ALS groups. One study of DEGs in the muscle of ALS patients, healthy controls, and ALS mimic disease patients, suggested that Myosin Binding Protein H (MyBP-H) could be a useful biomarker [124]. Gene expression profiling of *G86R* mice was able to detect a significant change in muscle gene expression prior to any obvious signs of motor neuron degradation [125]. Gene coexpression analysis on muscle tissue in ALS has yet to be reported. A study investigating differential gene coexpression between healthy and Duchenne muscular dystrophy (DMD), utilizing gene expression data from muscle biopsies, highlighted a possible utility of gene coexpression in ALS. The study found that when comparing healthy gene coexpression modules to DMD gene coexpression modules, preservation analysis could identify dysregulated processes in DMD [126]. In particular, new genes linked to the known pathological DMD gene were identified, expanding knowledge of the known disease pathway. This approach could serve to identify dysregulated processes in ALS muscle compared to healthy controls.

Proteomic analysis of ALS skeletal muscle has identified dysregulated protein quantities between ALS muscle and healthy controls, for example in the wobbler mouse model, which identified 21 upregulated proteins and 3 downregulated proteins involved in contractile apparatus and cell stress response [127]. The proteome of an ALS muscle biopsy sample may be influenced by denervation of the muscles from the MNs. As mentioned above, PPI networks represent an important resource for MIN approaches to the analysis of proteomic data. These have not yet been applied to ALS proteomic datasets, but have been used in studies of muscle pathology. For example, one study created a PPI network from 13 proteins and closely related proteins known to be dysregulated in limb girdle muscular dystrophies (LGMDs). A PPI network was constructed based on yeast 2 hybrid screening for the identification of binary protein interactions [128]. The PPI network was able to detect four novel proteins, ACTN2, MYBPC1, MYOM1, and MYOM2, identified as hub proteins sharing the highest number of links with known LGMD proteins. These novel proteins are located in key locations in the sarcomere, suggesting a link between LGMD-associated proteins and sarcomere dysfunction. Building a PPI network focused on ALS-associated proteins in muscle could help identify novel proteins involved in dysregulated processes in ALS.

### 4.2. Astrocytes

Numerous tissues and processes have been implicated in ALS pathology. One of the more widely studied cell types is the astrocyte glial subtype. Astrocytes play a pivotal role in the function of the central nervous system (CNS), with integral roles in the formation, maintenance, and elimination of synapses [129]. ALS-associated misfolded proteins SOD1 and TDP-43 have been identified as aggregating in glial cells, disrupting normal physiological function [43,130]. Astrocytes are implicated in numerous other neurodegenerative diseases, indicating their significance in neuronal pathology [131]. In an Alzheimer’s study, the astrocyte transcriptome was analysed in terms of the aging brain [132]. If a “healthy” control astrocyte transcriptome could be defined, identifying DEGs between control astrocytes and ALS astrocytes could aid in the understanding of the role of astrocytes in ALS. Astrocyte-specific datasets such as transcriptomic and proteomic profiling could be used in MIN approaches to ALS, and tissue-specific data on gene or protein expression could facilitate astrocyte-specific MIN analyses.

### 4.3. Mitochondria

Mitochondrial dysfunction has long been associated with ALS pathology, particularly in the *SOD1* mutation [133]. Many mechanisms of mitochondrial involvement in ALS have been identified, including clearance of dysfunctional organelles, calcium binding, and induction of mitochondrial death [134]. Gene expression profiling of iPSC-derived MNs from ALS fibroblasts has identified DEGs involving mitochondrial processes [135].

The transcriptome of the “healthy” mitochondria has previously been analysed [136]. Establishing DEGs between control and ALS mitochondria could provide an insight into their specific abnormalities in ALS. It would be desirable to test muscle and neuronal mitochondrial samples separately, due to tissue-specific mitochondrial gene expression. Previous studies have shown that it is possible to identify mitochondrial DEGs from blood samples [137,138,139]. Identifying DEGs can allow for the implementation of gene coexpression network analysis. This could elucidate mitochondrial and astrocyte processes specifically dysregulated in ALS. Furthermore, constructing a unique PPI network for known mitochondrial proteins, utilizing high throughput yeast 2 hybrid methods, could allow for dysregulated processes in ALS mitochondria to be predicted [140].

## 5. Conclusions

Personalized medicine provides the opportunity for improved treatment strategies in disease. Utilizing omics data is vital in establishing biomarkers for improved diagnosis, prognosis, and therapeutic targets. Molecular interaction networks represent a powerful aid in the analysis of omics data. ALS is a severe and fatal neuromuscular disease, and the lack of truly effective treatment underlines the need to incorporate cutting-edge approaches to discover improved therapeutics. Currently in ALS research, vast amounts of omics and other experimental data are being generated. However, the application of MINs is minimal. This review has highlighted MIN approaches in tissues involved in ALS that could provide greater insight into the pathological processes at play in ALS. The application of these approaches not only to motor neurons but also to other cell types such as muscle and astrocytes, or to specific subcellular structures such as mitochondria, should be a priority to help refine our understanding of underlying cellular molecular mechanisms in ALS.

Asides from approaches using MINs, which have been the subject of this review, several studies have applied mathematical modelling to attempt to understand the pathology in terms of relationships between high-order disease concepts such as “genetic damage”, “necro-apoptosis”, “calcium homeostasis”, “cellular respiration”, and other processes [141,142], whereas others have mathematically modelled clinical features in order to better predict disease progression in each patient [143,144]. MIN approaches may ultimately feed into higher order and clinical models, for example if they are used to better define molecular pathways or molecular biomarkers.

At the molecular level, the increasing availability of high-throughput data from experimental models and from large-population genomic and functional genomic studies, together with the growth of molecular interaction datasets and the optimization of network-clustering algorithms, may provide an opportunity to take a fresh look at the mechanistic processes underlying cellular pathology in ALS. Molecular changes at the level of network modules could be described per individual, lending itself to personalized medicine machine-learning approaches to model the relationships between dysregulated pathways. The clarification of pathological processes involved in ALS will guide the identification of biomarkers and potential drug targets in the future.
